# Supplementary material for: Gene flow biases population genetic inference of recombination rate
Source: G3 (Bethesda). 2022 Sep 14;12(11):jkac236. doi: 10.1093/g3journal/jkac236 (PMC9635666; doi:10.1093/g3journal/jkac236)
Supplement: jkac236_Supplementary_Data [file jkac236_supplementary_data.docx]

## Supplemental Material *For: “Gene flow biases population genetic inference of recombination rate” K. Samuk & M.A.F. Noor, G3, 2022.*


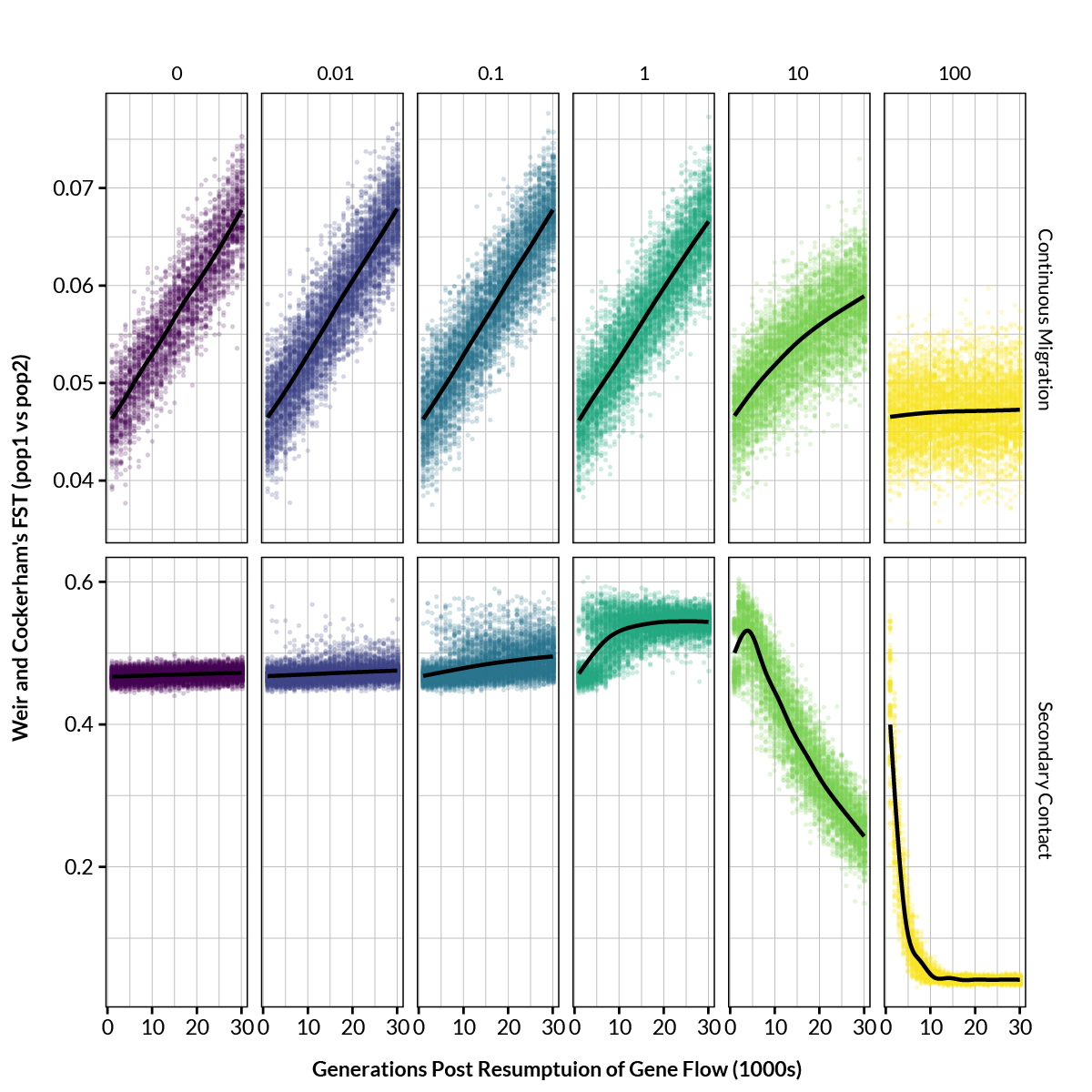


Figure S1 | Weir and Cockerham’s FST between simulated populations as a function of time in generations under various combinations of migration rate (columns, N_e_m) and isolation scenario (rows). Black lines are smoothed LOESS fits. Note the difference in y-axis scales between the rows.


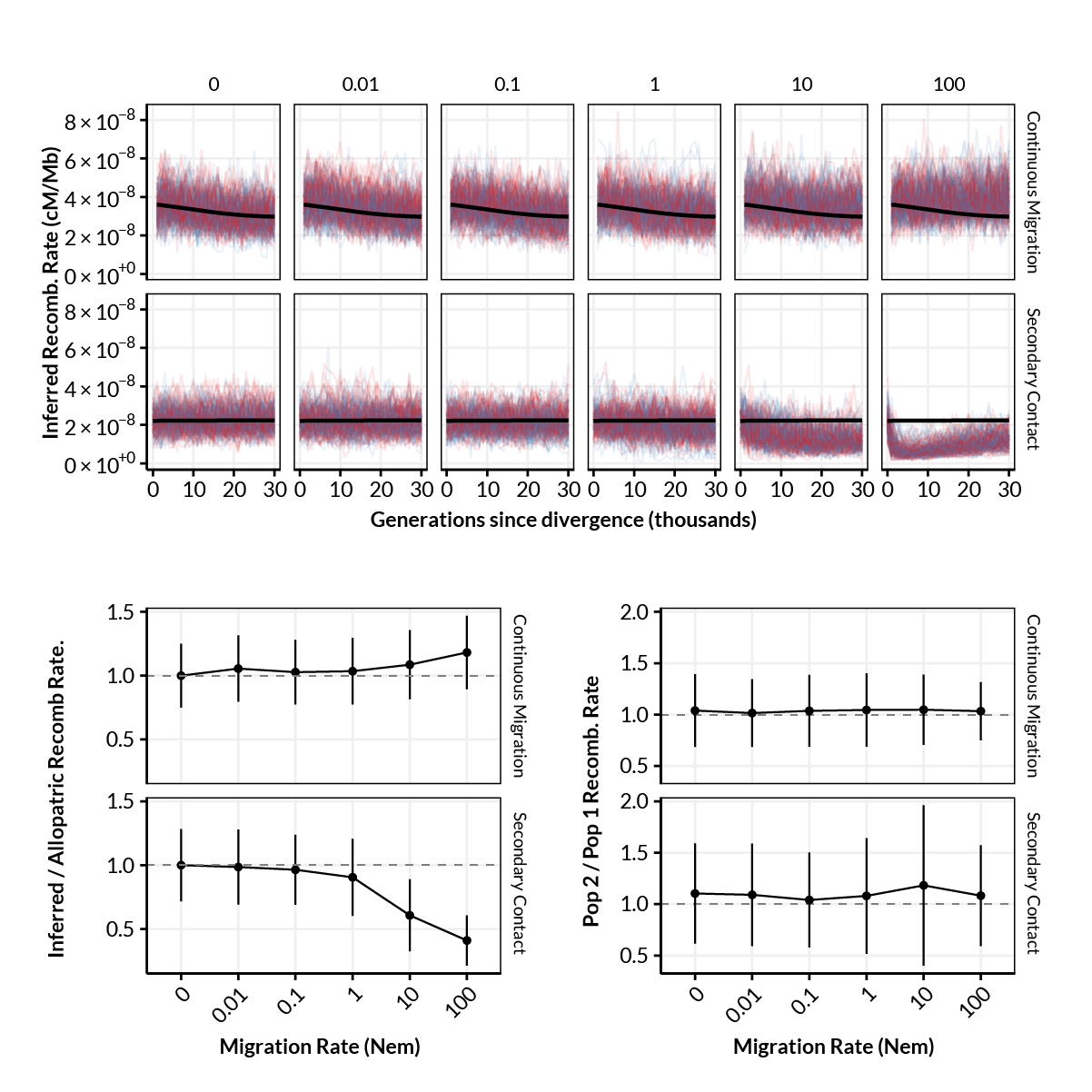


Figure S2 | The relationship between inferred recombination rate and the migration rate in simulated populations with Ne = 1720 where recombination rate remains constant in both subpopulations. (A) Inferred recombination rates for individual simulations at varying levels of migration. Each plot shows inferred rates for simulation replicates (transparent lines) of population 1 (red, unchanged recombination) and population 2 (blue, increased recombination) for a single migration rate. Dashed lines show the expected inferred value in the absence of gene flow (inferred from N_e_m = 0). (B) Summarized inferred recombination rates (y-axis) for each level of migration (x-axis) from the simulations in A. Points are mean values and error bars depict standard deviations (summarized across all generations). Dashed lines show the expected inferred value in the absence of gene flow for each population (i.e. the mean value for N_e_m = 0). (C) The inferred *difference* in recombination rate between population 1 and population 2 (*p_2_* - *p_1_*) as a function of migration rate. Points and errors bars are as in B.


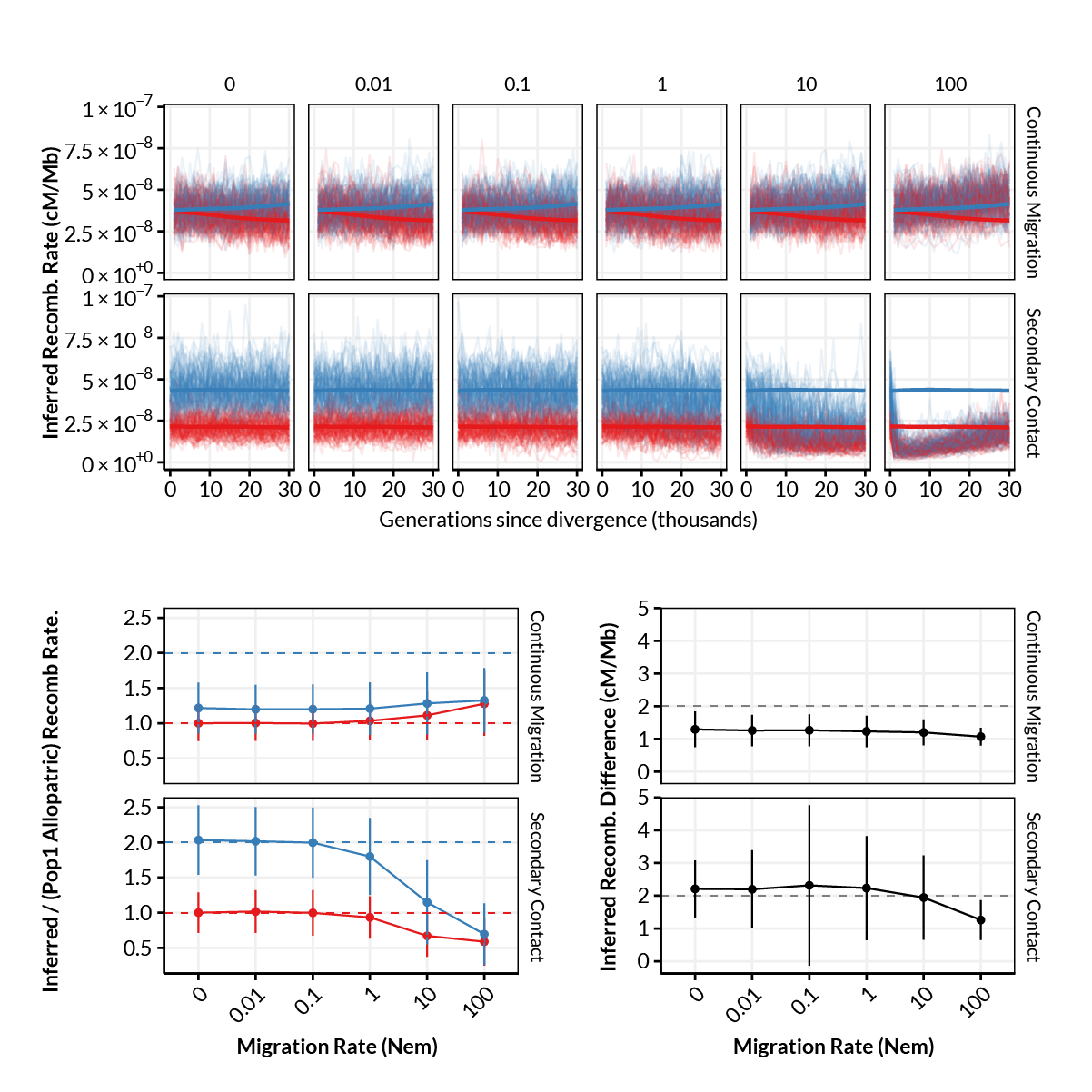


Figure S3 | The relationship between inferred recombination rate and the migration rate in simulated populations with Ne = 1720 where recombination rate increases by a factor of two in one subpopulation. (A) Inferred recombination rates for individual simulations at varying levels of migration. Each plot shows inferred rates for simulation replicates (transparent lines) of population 1 (red, unchanged recombination) and population 2 (blue, increased recombination) for a single migration rate. Dashed lines show the expected inferred value in the absence of gene flow (inferred from N_e_m = 0). (B) Summarized inferred recombination rates (y-axis) for each level of migration (x-axis) from the simulations in A. Points are mean values and error bars depict standard deviations (summarized across all generations). Dashed lines show the expected inferred value in the absence of gene flow for each population (i.e. the mean value for N_e_m = 0). (C) The inferred *difference* in recombination rate between population 1 and population 2 (*p2* - *p_1_*) as a function of migration rate. Points and errors bars are as in B.


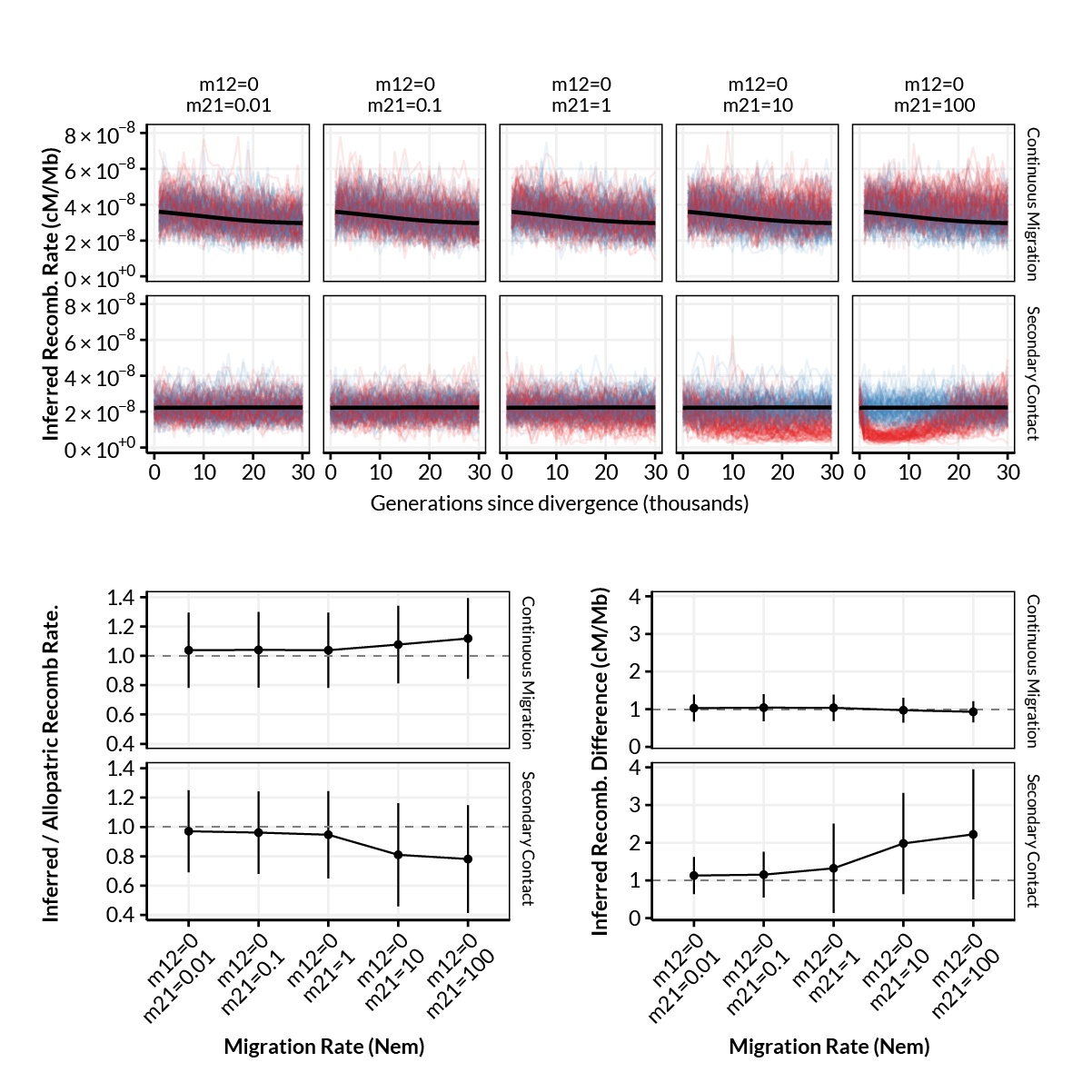


Figure S4 | The relationship between inferred recombination rate and the migration rate in simulated populations with Ne = 1720 where recombination rate remains constant in both subpopulations and gene flow is unidirectional (p2 to p1 only). (A) Inferred recombination rates for individual simulations at varying levels of unidirectional gene flow. Each plot shows inferred rates for simulation replicates (transparent lines) of population 1 (red, unchanged recombination) and population 2 (blue, increased recombination) for a single migration rate. Dashed lines show the expected inferred value in the absence of gene flow (inferred from N_e_m = 0). (B) Summarized inferred recombination rates (y-axis) for each level of migration (x-axis) from the simulations in A. Points are mean values and error bars depict standard deviations (summarized across all generations). Dashed lines show the expected inferred value in the absence of gene flow for each population (i.e. the mean value for N_e_m = 0). (C) The inferred *difference* in recombination rate between population 1 and population 2 (*p_2_* - *p_1_*) as a function of migration rate. Points and errors bars are as in B.


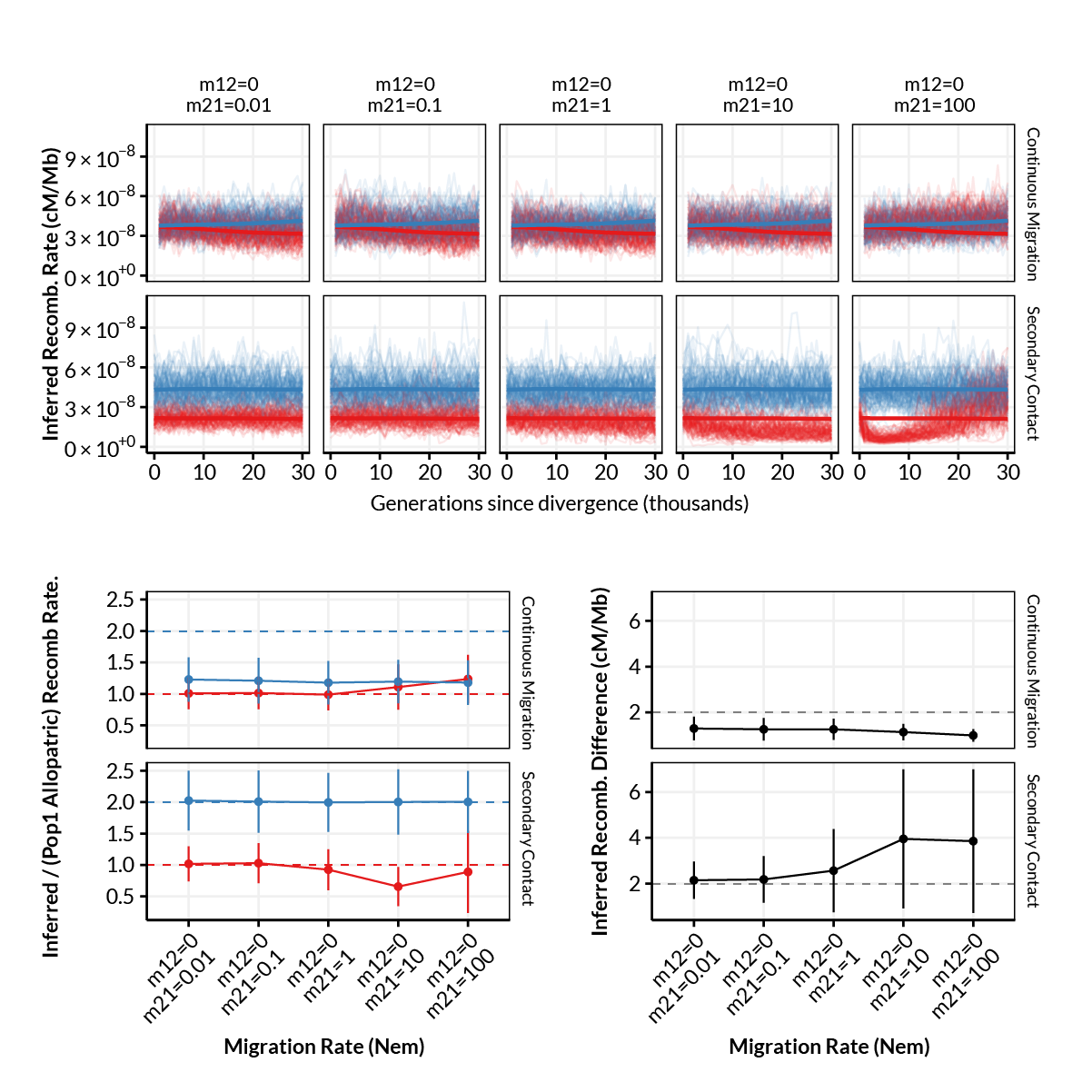


Figure S5 | The relationship between inferred recombination rate and the migration rate in simulated populations with Ne = 1720 where recombination rate increases by a factor of two in one subpopulation and gene flow is unidirectional (p2 to p1 only). (A) Inferred recombination rates for individual simulations at varying levels of unidirectional migration. Each plot shows inferred rates for simulation replicates (transparent lines) of population 1 (red, unchanged recombination) and population 2 (blue, increased recombination) for a single migration rate. Dashed lines show the expected inferred value in the absence of gene flow (inferred from N_e_m = 0). (B) Summarized inferred recombination rates (y-axis) for each level of migration (x-axis) from the simulations in A. Points are mean values and error bars depict standard deviations (summarized across all generations). Dashed lines show the expected inferred value in the absence of gene flow for each population (i.e. the mean value for N_e_m = 0). (C) The inferred *difference* in recombination rate between population 1 and population 2 (*p2* - *p_1_*) as a function of migration rate. Points and errors bars are as in B.
